# Star Can Vac Capsulorhexis in White Total Cataracts - A Retrospective Interventional Analysis

**DOI:** 10.22336/rjo.2024.73

**Published:** 2024

**Authors:** Kodavoor Shreesha Kumar, Raju Sumithra, S. Tamilarasi, Dandapani Ramamurthy

**Affiliations:** 1 The Eye Foundation, Coimbatore, Tamil Nadu, India

**Keywords:** Star CanVac, intumescent cataract, vacuum rhexis, IOL = Intraocular lens, CCC = continuous curvilinear capsulorhexis, BSS = Balanced salt solution, SICS = Small Incision Cataract Surgery, CME = Cystoid Macular Edema, OVD = Ophthalmic Viscoelastic Device

## Abstract

**Aim:**

Completing circular uniform anterior capsulorhexis in intumescent white cataracts is challenging for all cataract surgeons. Numerous techniques have been described to get a circular capsulorhexis and prevent perpendicular linear tears in the anterior capsule.

**Methods:**

570 cases of white total cataracts were selected for this retrospective clinical study. In this technique of Star CanVac rhexis, the anterior lens capsule was nicked using a 26 G cystotome, and multiple centripetal tears were made in the center to create a small star-shaped opening. A vacuum was used to develop capsulorhexis, and a 25 G flat tip cannula attached to a 5 ml syringe half filled with balanced salt solution (BSS) was used to build capsulorhexis. The piston of the 5 ml syringe was withdrawn to create a vacuum to hold the free capsular flap. It was then directed circularly to get a round capsulorhexis. Oozing liquefied cortex was aspirated simultaneously with the same cannula.

**Results:**

This technique was successfully executed in 564 eyes. Six eyes had anterior capsular tears, 2 of which extended into the posterior capsule.

**Discussion:**

Intumescent cataracts often complicate the rhexis procedure due to increased lens volume and pressure. Over time, different methods have been refined to handle the pressure variation between the anterior chamber and the intralenticular area, such as mini-rhexis, double rhexis, sewing needle capsulotomy, and phaco capsulotomy. The primary goal of these procedures is to first reduce the elevated intralenticular pressure. Star CanVac capsulotomy facilitates equal pressure between the anterior chamber and the lenticular compartment, effectively reducing the risk of accidental capsular tears. Advantages of this approach include completing rhexis in one step, removing the liquefied cortex simultaneously, and relying on easily accessible instruments.

**Conclusion:**

Star CanVac capsulorhexis is an effective, safe, and alternative technique to conventional capsulorhexis in total white cataracts.

## Introduction

Cataract surgery has progressed from a vision restorative procedure to refractive surgery. Well-centered capsulorhexis and in-the-bag intraocular lens placement (IOL) are imperative to have good visual outcomes and stabilize the capsular bag during hydro procedures and nucleus and cortex removal. Intumescent cataracts have high intralenticular pressure due to fluid accumulation in a liquefied cortex or viscous cortex, making the anterior capsule tenser. Standard capsulorhexis with 26 G cystotome in such cataracts increases the risk of capsular flap extension to the equator and subsequently to the posterior capsule, resulting in poor outcomes due to complications like vitreous loss, nucleus drop, and aphakia. Any surgical technique that decompresses the intralenticular pressure before propagating the tear decreases the risk of anterior capsular tear. Kodavoor et al. [1] published a new manual technique of Star CanVac continuous curvilinear capsulorhexis (CCC) in the year 2022 for doing capsulorhexis in intumescent cataract using a 26 G cystotome bent at the tip, a 25 G flat-tipped fine cannula connected to a 5 mL syringe half filled with balanced salt solution (BSS). In this study, we analyzed the success of the same technique in an extensive series of total white cataracts.

## Methods

Ethics committee approval was obtained, and the study adhered to the tenets of the Declaration of Helsinki. Informed consent was obtained from the patients. The study period was between 2017 and 2023. Patients with mature white cataracts were included in the study. Traumatic cataracts, uveitic cataracts, and eyes with capsular fibrosis or zonular weakness were excluded from the study.

Preoperative cataract workup included complete refraction, intraocular pressure measurement by Goldmann applanation tonometry, comprehensive slit lamp examination (presence of fluid aperture within the lens fibers of the white cataract with corresponding shallow anterior chamber was noted, and white cataracts were classified as intumescent or non-intumescent white total cataract) and B Scan ultrasonography for posterior segment examination. Biometry was performed using IOL Master 700 (Zeiss, Germany) or ultrasound-guided immersion A-scan (Occuscan, RxP, Alcon Laboratories, Inc.) whenever the IOL master could not calculate axial length. IOL power calculation was done using the Barrett Universal II formula.

Preoperatively, the operating eye was dilated with Tropicamide 1.0% and phenylephrine 2.5% eyedrops. A single surgeon performed all the surgeries. Phacoemulsification was performed through a temporal 2.2 mm precise corneal incision under peribulbar anesthesia (using 2.0% Xylocaine and 0.75% Bupivacaine). Two side ports were created using an MVR blade. Under an air bubble, anterior capsular staining was done with trypan blue 0.06% (Auroblue, Aurolabs, India). An ophthalmic viscoelastic device (OVD) was (Appavisc, Appasamy, India) injected to form and deepen the anterior chamber. Sodium hyaluronate 1.4% (Aurogel, Aurolabs, India) flattened the anterior lens capsule wherever required. 26 G bent cystotome needle was used to create five to six small tears over the center of the anterior lens capsule (trim star-shaped configuration) in a centripetal direction. These small star-shaped multiple capsular tears decompress the capsular bag and equally distribute the forces into torn capsular margins to prevent spontaneous, unidirectional, or bidirectional, peripheral extension of capsular tears.

**Fig. 1:**
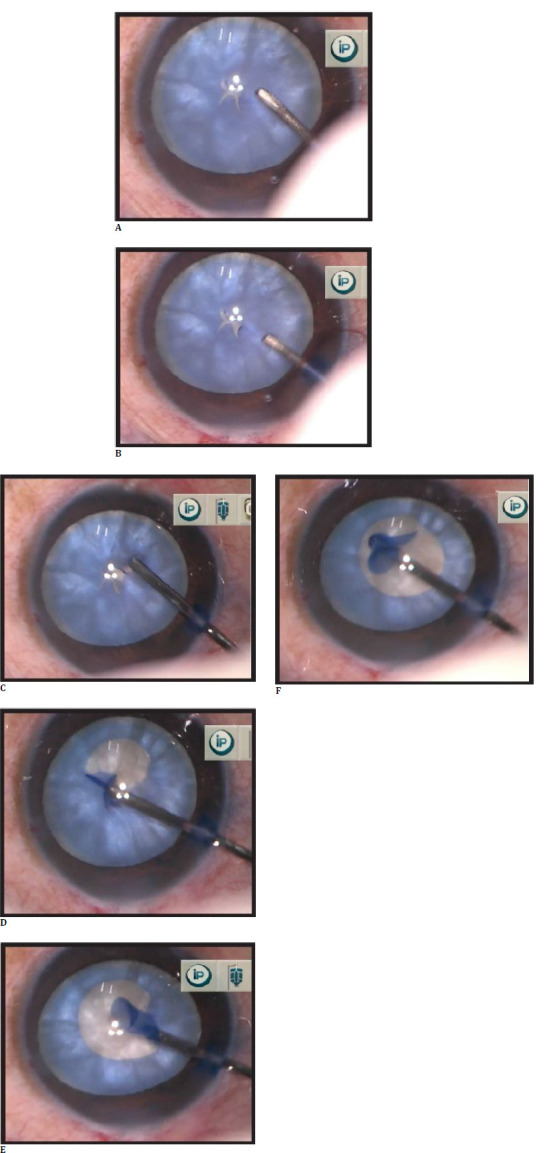
**A**. Intumescent white cataract with anterior lens capsule stained with trypan blue - Multiple small tears are created in the capsule's center in a star-shaped configuration with seepage of milky fluid; **B**. Anterior chamber filled with OVD; **C**. One of the flaps is vacuumed with a 25 G flat-tipped cannula; STAR CanVac rhexis is initiated; **D**. Propagation of star CanVac rhexis in a circular fashion; **E**. Flap re-grasped whenever needed to continue rhexis; **F**. Completed STAR CanVac rhexis.

Subsequently, a 25 G flat-tipped cannula attached to a 5 ml syringe half filled with BSS was used to hold free capsular flaps by suction pressure, and a vacuum was created by withdrawing the syringe’s piston. It was maneuvered in a circular motion to get a round capsulorhexis (**Fig. 1 A-F**). To achieve reasonable control during capsulorhexis, the capsular flap can be grasped and re-grasped several times by releasing the vacuum, which was done by pushing the piston gently. Simultaneously, the loosened and liquified cortex can be aspirated with the same cannula, which aids in better visualization and reduces intracapsular pressure.

## Results

Star CanVac capsulorhexis was carried out in 570 eyes of 545 patients with total white cataracts. Demographic details of study participants and study period are mentioned in **Table 1**. Among 570 eyes, 442 (77.54%) had an intumescent type, and 128 (22.45%) had nonintumescent mature white cataracts. Of these, unilateral cataract was noted in 520 patients and bilateral in 25 patients, with a male-to-female ratio of 301: 244. The follow-up period was 6 months. Double and triple rhexis were done using utrata/microrhexis forceps in 91 (15.96%) and 12 (2.10%) eyes, respectively. Rhexis enlargement (for irregular, asymmetric rhexis) was required in 44 (7.71%) eyes after IOL implantation to prevent postoperative capsular phimosis.

**Table 1:** Demographic data of the study subjects and their follow-up period

Demographic data	Number of patients with mean age and Duration of study
Number of patients	545
Total number of cases	570
Unilateral: bilateral	520:25
Male: female	301:244
Mean age	51 years (range: 27-76 years)
Average follow-up period	6 months

**Table 2** represents the results of the Star Can Vac technique. Except for 6 (1.05%) eyes, all eyes had successful capsulorhexis with this technique. Inadvertent anterior capsular tear happened in these 6 cases with an intumescent type of white cataract. Phacoemulsification was continued and completed carefully with due precautions (slow motion phaco while maintaining chamber stability) in 3 eyes with anterior lens capsule tear. In 3 eyes, we converted it into manual minor incision cataract surgery (SICS), of which 2 (0.35%) eyes had posterior capsular tear with vitreous prolapse. Anterior vitrectomy was done to remove the prolapsed vitreous. A multipiece hydrophobic intraocular lens was placed in the sulcus in two eyes, which had adequate sulcus support, and an iris clip IOL was placed in one eye, which lacked sulcus support. One eye developed cystoid macular edema (CME) in the third month of the postoperative visit, for which an intravitreal dexamethasone implant (cross consultation with Vitreoretinal colleague taken) was given, and CME resolved.

**Table 2:** Outcomes of Star Can Vac capsulorhexis in total white cataracts

Results	Number of eyes (%)
**Type of total cataract**	
Intumescent cataract Non-intumescent cataract	442 (77.54%) 128 (22.45%)
**Surgical method**	
Phacoemulsification MICS (conversion due to rhexis extension)	567 (99.47%) 3 (0.52%)
**Complications**	
Anterior capsular tear Posterior capsular tear Anterior vitrectomy Cystoid macular edema	6 (1.05%) 2 (0.35%) 2 (0.35%) 1 (0.17%)
**IOL placement**	
In capsular bag Sulcus Iris clip IOL	567 (99.47%) 2 (0.35%) 1 (0.17%)

## Discussion

Jacobs, Agarwal, et al. [2] reported an incidence of incomplete capsulorhexis in white cataracts, which ranged from 3.85% to 28.3%. Gimbel and associates discovered the shearing and tearing concept in circular curvilinear capsulorhexis in cataract surgery, which became the traditional method in immature cataracts.

Vitreous upthrust was believed to be one of the causes of capsulorhexis extension in intumescent cataracts. Preoperative use of intravenous mannitol was thought to counteract the positive vitreous pressure, even with its use of rhexis extension was reported in many patients. Thus, this hypothesis has no corroboration to support its definitive role in intumescent cataracts. Figueiredo CG et al. [3] postulated the presence of anterior and posterior intralenticular pressure in intumescent cataracts. More fluid accumulated between the posterior capsule and nucleus, which led to high posterior intralenticular pressure and provoked the capsular tear extension during the initiation of rhexis.

Multiple techniques have evolved to tackle the pressure gradient between the anterior chamber and the intralenticular compartment. Cohesive OVD helps to flatten the anterior lens capsule and helps to propagate rhexis in intumescent white cataracts. Nabil KM [4] suggested a lens decompression technique using a 25 G needle to aspirate the liquified cortex by puncturing the center of the anterior lens capsule. Kara-Junior N et al. [5] and Ucar F [6] et al. devised mini rhexis and spiral capsulorhexis in intumescent cataracts. In both these methods, a smaller-sized rhexis was done initially, after which lens decompression was done by removing the cortex. This was followed by enlarging the previously made Mini rhexis for nucleus management. Teng C et al. [7] propounded a phaco capsulotomy technique using a phaco probe to create anterior capsulotomy and lens decompression. Irregular capsular margin, radial extension, wound burn, and zonular weakness were the expected complications from that procedure. In Prasad et al.’s sewing needle capsulotomy technique [8], a unique micro capsulotomy needle produced a smooth round opening instead of a linear cut by a hypodermic needle to prevent the Argentinian flag sign. More advanced technologies like Femtosecond laser-assisted capsulotomy and Zepto precision pulse capsulotomy can be used in intumescent cataracts to achieve precise-sized capsulotomy. Ununiform laser delivery due to leaked fluid, high cost, and limited availability were limitations of those technologies.

Star CanVac capsulotomy is the upgraded version of our technique of CanVac capsulorhexis [9], with the added step of making a star-shaped capsular tear to achieve equal pressure between the anterior chamber and lenticular compartment, which further minimizes the chance of accidental capsular tears. Selecting an appropriate cannula size is essential. A small-sized bore can cause inadequate suction, while a more significant size can cause flap amputation and anterior chamber collapse by removing a large amount of OVD. Considering this, we used a 25 G cannula.

Complete capsulorhexis can be done as a single-staged procedure without coming out of the anterior chamber (this prevents collapse of the anterior chamber and hence rhexis extension). Using the same cannula to visualize the rhexis margin, we can also aspirate the liquefied cortex. Our technique’s advantage is using a readily available instrument like a 25 G cannula, which gives reasonable control during capsulorhexis.

## Conclusion

STAR CanVac can be considered a better alternative to conventional capsulorhexis in white cataracts. It takes a few cases to learn, and it can be mastered by every surgeon in any circumstance, both in phacoemulsification and manual SICS.
